# Air stable black phosphorous in polyaniline-based nanocomposite

**DOI:** 10.1038/s41598-017-10533-5

**Published:** 2017-08-31

**Authors:** Jéssica E. S. Fonsaca, Sergio H. Domingues, Elisa S. Orth, Aldo J. G. Zarbin

**Affiliations:** 1 Department of Chemistry, Federal University of Paraná (UFPR), CP 19032, CEP 81531-980, Curitiba, PR Brazil; 2 Graphene and Nano-materials Research Center – Mackgraphe – Mackenzie Presbyterian University, 01302-907 São Paulo, Brazil

## Abstract

The greatest challenge regarding black phosphorus (BP) comes as a result of its fast degradation when exposed to ambient conditions, which has overshadowed its applications. Herein, we report a simple and efficient route towards overcoming BP deterioration by preparing a nanocomposite with the conducting polymer polyaniline (PANI). The liquid/liquid interfacial method was employed to produce transparent, freestanding and transferable thin film of BP covered by PANI, with high stability under ambient atmosphere, up to 60 days. Otherwise, the uncapped exfoliated neat BP degraded in solely 3 days under the same conditions. Characterization data show that PANI covers efficiently the BP flakes, indicating favorable interactions between the components. The results presented here can be considered a breakthrough for employing BP as thin film in different technological applications, considering the properties of BP itself or taking advantage of synergistically combining the properties of both components.

## Introduction

Black phosphorus (BP) has risen as a physical analogue of graphite, with orthorhombic puckered layers^1^ consisting of P atoms covalently bonded to three neighbors^2^. Since its first exfoliation in 2014^3^, BP mono- or few layers has shown intriguing potential applications due to a wide range of interesting properties, such as a tunable band gap with thickness^4^, high charge mobility (up to 1000 cm^2^/V s)^3^ and anisotropic transport^4^. Although these features bring new and exciting possibilities, the instability of BP against air has deeply limited its synthesis and processing, preventing the development of BP-based devices and electronics^5^. Recently, it has been demonstrated that the reaction with oxygen governs the degradation of pristine BP^6^ and may be photoactivated by the presence of aqueous oxygen^5^, thus suggesting that water does not play a determining factor^6^. The degradation of BP causes physical and chemical changes in the material, resulting in air-degraded products with no defined stoichiometry^7^. According to recent studies, possible degradation products could be ascribed to phosphorus oxides^8^ or, as suggested by the previous work of Bridgman, as phosphorus oxyacids^9^.

As matter of paramount significance for future applications, many research efforts have been made in order to find a way out to air-stable BP devices. In fact, two effective ways have been employed for this purpose: (i) solvent exfoliation^10, 11^, which results in a solvation shell that protects BP surface against the approach of oxidative species and (ii) passivation through encapsulation with other materials, such as graphene^12^, boron nitride^13^, aluminum oxide layers^8^ and polymers (*e*.*g*. poly(methyl methacrylate))^14^. Although these methods are well accepted as alternatives to stabilize BP, they have some drawbacks concerning large scale production, high costs and lack of flexibility in processing and depositing the resulting material. Besides, the encapsulation through the simple sandwiching of BP does not extent the materials properties by means of synergistic effects. Thus, the production of nanocomposites of BP and other materials could be an interesting alternative to obtain stabilized materials with enhanced properties. In this sense, we highlight the class of conducting polymers, such as polyaniline (PANI), which is commonly employed as a anti-corrosion coating of metals^15^. Thereby, PANI comprises a promising candidate to provide enhanced stability for BP leading to optimized nanocomposites with synergistic effect due to the outstanding electrical, electrochemical, optical, magnetic and mechanical properties of PANI^16^.

Our research group has developed a novel route for the synthesis of different conducting polymer-based nanocomposites^17–19^, in which a highly stable, homogeneous and free-standing film is formed at a liquid-liquid (L/L) interface, easily to be deposited over any desirable substrate^18^. Inspired in obtaining stabilized BP-based films, we developed a novel route to nanocomposites of BP/PANI as thin films. Aniline was polymerized directly in an exfoliated BP dispersion, leading to a polymeric nanocomposite with BP sheets strongly covered by PANI. The stability of the obtained material was evaluated by spectroscopic techniques and compared to the uncapped material. Indeed, we show that the polymer suppresses the chemical degradation of exfoliated BP, which lasts up to 60 days under ambient exposure that degrades in solely 3 days for bare BP. To the best of our knowledge, there are no such systematic works assessing the stability of BP incorporated into polymeric matrices^14, 20^ just as there are no descriptions regarding the synthesis, processing and characterization of a BP/PANI thin film nanocomposite.

## Methods

### BP exfoliation

**Figure 1:**
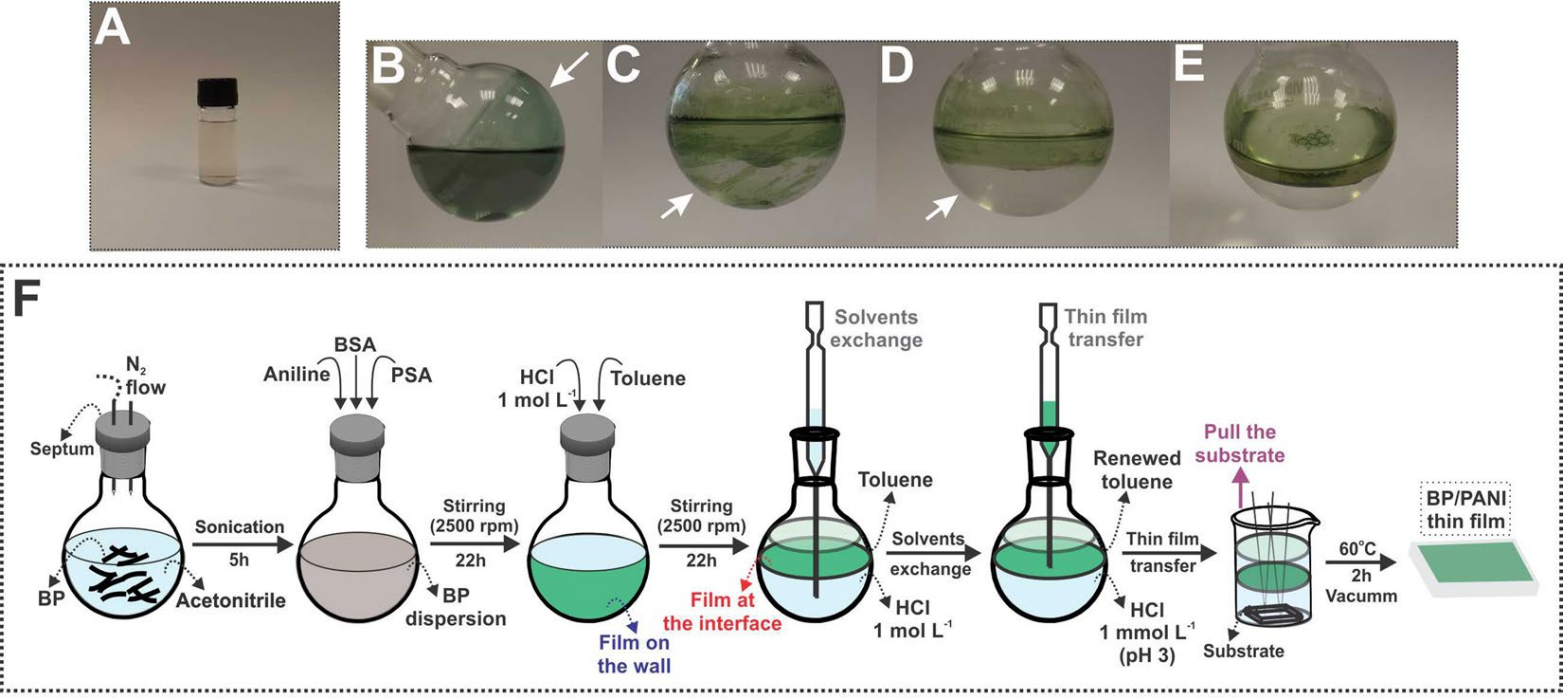
Digital photographs of (**A**) BP dispersion in acetonitrile along with the reaction flask for BP/PANI formation showing the film: (**B**) attached to the wall, (**C**) detaching from the wall and (**D**,**E**) established at the L/L interface. The schematic representation (**F**) shows the overall steps for the preparation of BP/PANI thin film.

0.5 mg of BP crystals (Smart Elements) was added to a 50 mL sealed round-flask containing 20 mL of acetonitrile previously purged with N_2_ flow (5 min). The material was exfoliated through the sonication in an ultrasonic bath (37 kHz) at 20 °C for 5 h under continuous N_2_ flow that resulted in a brown dispersion (Fig. 1A).

### Nanocomposite preparation

Figure 1B–E shows photographs during the reaction for the formation of the BP/PANI nanocomposite that is schematically summarized in Fig. 1F and described in detail in the following^21^. Firstly, 10 μL of aniline was added to the flask containing the BP dispersion, followed by the addition of 6.4 mg of ammonium persulfate (PSA) and 35.4 mg of benzenesulfonic acid (BSA). After around 20 min, a blue color appears, followed by a green color typical of emeraldine salt (ES), the conducting form of the polymer. Thus, by the end of 22 h of stirring (2500 rpm), a green solid was observed adhered to the flask wall, as indicated by the arrow in Fig. 1B. In the following, the acetonitrile (with very few solid remnants) was removed and discarded, and an interfacial system containing 20 mL of an aqueous solution of HCl (1 mol L^−1^) and 10 mL of toluene was added to the same flask. Then, after the mixture stirred for 22 h (2500 rpm), the green solid migrated spontaneously from the flask wall to the L/L interface (see arrows in Fig. 1C,D), resulting in the formation of a self-assembled green film (Fig. 1E). The next steps of this process involved the solvents exchange, which was performed with the aid of a pipette, as depicted in Fig. 1F, in order to remove excess acidity and side products from the polymerization. The toluene present in the organic phase was repeatedly changed by consecutive removals followed by the additions of new portions of the solvent (three times). In the same manner, the aqueous phase was partially removed and a solution of HCl 10^−3^ mol L^−1^ was added to the system, this process was consecutively repeated until pH 3. For the films deposition, a beaker (10 mL) containing the substrate of interest (glass, ZnSe crystal window, Si/SiO_2_ or quartz) at the bottom was prepared with the same solvent system present in the flask. The stabilized film was transferred to the beaker with the aid of a pipette (see Fig. 1F), which promptly stabilizes at the new L/L interface. In the following, the substrate was pulled towards the interface, depositing the nanocomposite film (BP/PANI). The material was vacuum dried for 2 h at 60 °C. For comparison purposes, we obtained control samples of both neat PANI and BP that were synthesized separately. The synthesis of neat PANI was carried out without the presence of BP, following the same process described above that lead to a stable film of at the L/L interface. The production of a pure BP film, however, was unfeasible. Although the exfoliated BP mostly concentrated at the L/L interface, no film was formed. Thus, the neat BP control sample was obtained by collecting an aliquot of the BP dispersion from the interface and drying over the desired substrate (60 °C, air-free atmosphere).

### Nanocomposite stabilization study

We monitored the nanocomposite exposed to ambient conditions (25 °C, 60% of humidity) for up to 60 days. The sample was named according to the exposure time (days): BP/PANI-0, BP/PANI-10, BP/PANI-15, BP/PANI-20, BP/PANI-30 and BP/PANI-60. The same monitoring was performed with the BP dispersion dried on the substrate (control sample of neat BP) being named according to the exposure time: BP-0, BP-3 and BP-15. The samples BP/PANI-0 and BP-0 mean that their exposure was suppressed to the maximum and characterizations were taken immediately after preparation. Overall, samples were maintained under the same storage conditions and periodically, Raman and FTIR spectroscopies analysis were taken (detailed parameters are depicted in the following).

### Characterization

Raman spectra and images were collected in a confocal Raman spectrometer (WITec Alpha 300 R) using a 532 nm laser line and 100× objective lens, with the samples deposited over a glass substrate. The optical power was kept at 1 mW in order to avoid laser-induced thermal effects. Fourier Transform Infrared spectra (FTIR) were carried out on a FTIR Bio-Rad spectrophotometer over the range of 4000–700 cm^−1^, using a ZnSe crystal window as substrate. Scanning Electron Microscopy (SEM) images were obtained using a Mira FEG-SEM TESCAN operated at an accelerating voltage of 10 kV with an In Beam detector. The X-ray Dispersive Energy (EDS) analyses were performed on an OXFORD detector coupled to the microscope. The samples were prepared by the direct deposition on Si/SiO_2_ substrates. UV–Vis spectra were obtained directly from the films deposited over quartz, in a Shimadzu UV- 2450 spectrophotometer.

## Results

### Characterization of exfoliated BP

Firstly, the liquid-phase exfoliation was used to produce BP sheets, knowingly a powerful technique to produce homogeneous BP dispersions in large quantities^10^. For this purpose, sonication of BP crystals was carried out in acetonitrile (air-free atmosphere), leading to a brown dispersion, expected for this material. In fact, acetonitrile has been reported as an efficient exfoliation solvent for BP^22^, besides it presents a lower boiling point than other suitable solvents reported^23^. Additionally, acetonitrile showed to be a good solvent for aniline polymerization, fundamental for the following steps of this work.

In order to validate the production of exfoliated BP, morphological and spectroscopic techniques were employed for its characterization, which were performed right after the sample preparation, and are given in the Supplementary Information (SI). The obtained Raman and Infrared spectra are shown in Fig. S1. Figure S1A depicts the presence of Raman bands centered at 363 cm^−1^, 440 cm^−1^ and 468 cm^−1^, corresponding to the vibrations of the material network, respectively attributed to the active modes ${{\rm{A}}}_{1{\rm{g}}}$, ${{\rm{B}}}_{2{\rm{g}}}$ e ${{\rm{A}}}_{2{\rm{g}}}$ ^24^, which confirms crystalline BP even after the exfoliation process^25^. The scheme shown in the inset indicates the direction of vibration of the phosphorus atoms in the different Raman modes. In addition, bands between 800 and 950 cm^−1^ (indicated by the asterisk in Fig. S1A) were observed and refer to the second order spectrum of BP^26^.

Fig. S1B shows the FTIR spectrum of exfoliated BP, evidencing the presence of some very low intense bands referring to modes resulting from the phosphorus oxidation process^27, 28^: P-OH bending at around 1060 cm^−1^ and P-O-P linkages stretching centered at 840 cm^−1^. In addition, other discrete bands are observed at around 1200 cm^−1^ (see asterisk in Fig. S1B) and could be related to the P-O stretching that sometimes appears as a doublet^29^. Moreover, the high noise observed in the 1650 cm^−1^ spectral region indicates the sample moistening^30^, which is expected in the BP degradation process. In fact, the coexistence of these modes of vibration in the exfoliated material show that the BP degradation results in the formation of P_x_O_y_ species, as previously described in the literature^5, 8^.

The morphology of the material was investigated by SEM and the obtained images, along with the corresponding EDS elementary mapping, are presented in Fig. S2. The SEM images confirm the production of exfoliated BP with lateral dimensions that reach tens of micrometers and vary considerably in the number of sheets. The EDS elemental map observed along with the images reveals a homogeneous distribution of the phosphorus over the flake, corroborating with the corresponding spectrum that presented only the peak referring to P. The Si peak refers to the substrate where the dispersion was deposited.

Thus, we have confirmed the nature of the exfoliated BP. In addition, even though characterization were taken immediately after sample preparation (with vacuum drying), there are evidences of oxidation, which agrees with the highly unstable nature of the material. Henceforth, we pursued the synthesis of the BP/PANI nanocomposite, as described in the following.

### Characterization of BP/PANI nanocomposite

BP/PANI nanocomposite was obtained through a simple and straightforward two-step method. Firstly, aniline was polymerized in a deaerated dispersion of the exfoliated BP in acetonitrile, exactly as described before, in order to have comparable samples. PANI grows covering the BP sheets and protecting against air degradation. The resulting material is sequentially transferred to a L/L biphasic system (aqueous HCl solution/toluene), leading to a BP/PANI nanocomposite thin film at the interface. For comparison purposes, we obtained control samples by submitting the exfoliated BP and aniline, independently, to the same process, thus seeking to obtain neat BP and PANI films. However, despite exfoliated BP concentrated at the interface, no film was formed, indicating that the presence of PANI is indispensable for the formation of a stable film of this material. Thus, we evaluated the PANI control sample as a thin film and the pure BP sample was collected from the interface and dropped and vacuum dried over different substrates according to the characterization analysis.

Fig. S3 presents the UV-Vis spectra of neat PANI and BP/PANI nanocomposites. Spectrum of the neat polymer, as well as of the nanocomposite, present all the typical bands of PANI in its conducting form, *i*.*e*. emeraldine salt (ES), confirming its formation: at 350 nm, related to transitions from the valence to the conduction band; at 450 nm, due to the transition from the polaronic to the conduction band and at 800 nm, attributed to the transition from the valence to the polaronic band^31^. As observed, the insertion of BP in the PANI structure does not cause any considerable optical or electronic changes in the material.

**Figure 2:**
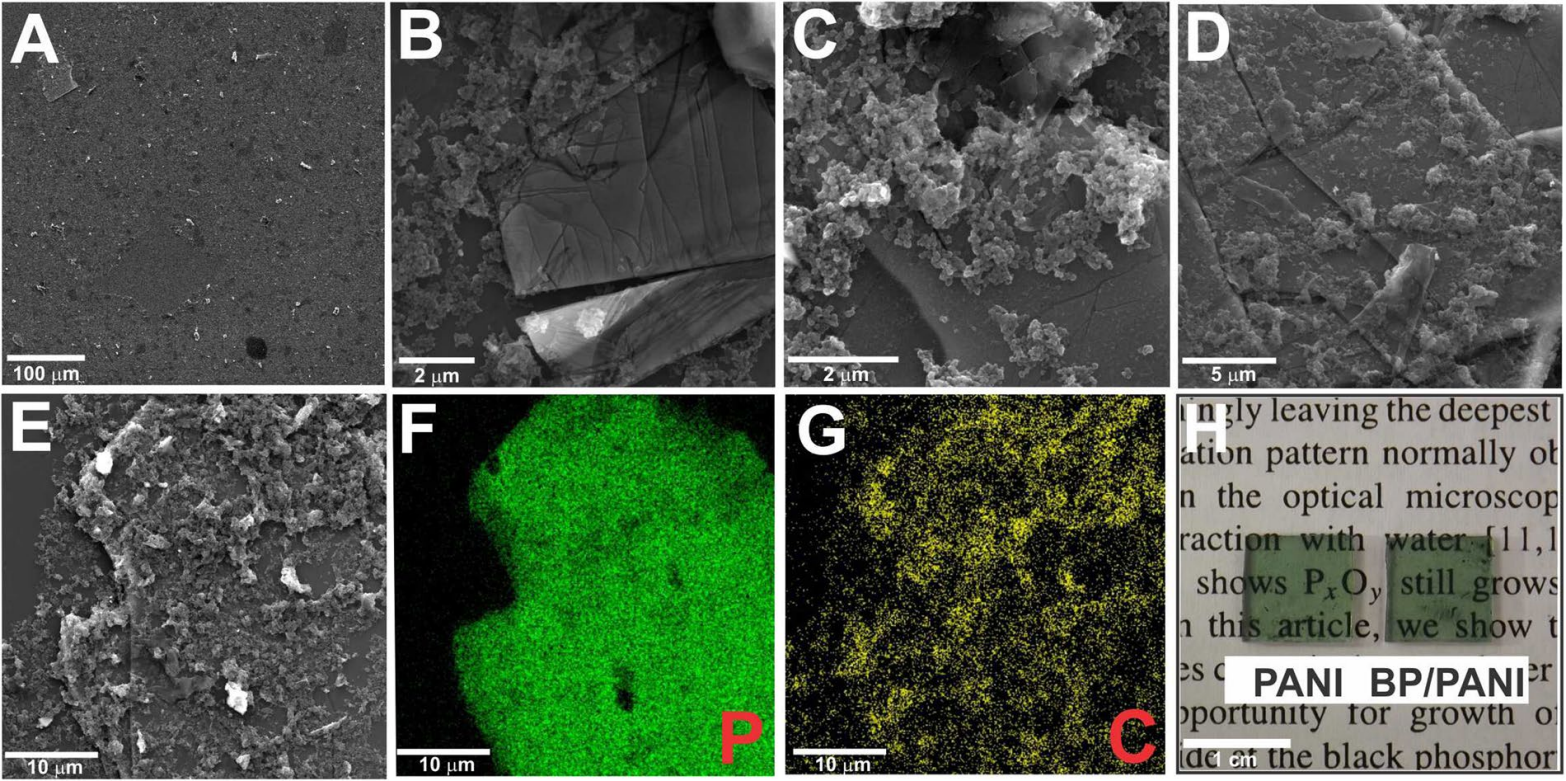
(**A**–**E**) SEM images of BP/PANI thin film in different regions of the sample and (**F**,**G**) elemental mapping, along with (**H**) digital photographs of the films PANI and BP/PANI deposited over glass taken over a text, evidencing transparency and optical quality.

SEM images are exhibited in Fig. 2 along with the digital photographs of the films. From Fig. 2A, at a lower magnification, it is possible to verify the uniformity of the film, which completely covers the substrate where it is deposited. Figure 2B–E clearly shows the morphological difference between the two components, in which BP appears as flat sheets and PANI as agglomerated flakes. It is evident from Fig. 2B–E that the nanocomposite presents portions in which BP is partially (Fig. 2B,C) or completely (Fig. 2D,E) covered by the polymer. The elemental mapping performed by EDS (Fig. 2F,G) from the image presented in Fig. 2E shows the homogeneous distribution of both P and C over the entire region analyzed, indicating a favorable interaction between the components. Furthermore, the green color of the obtained films, exhibited in the photographs shown in Fig. 2H, corroborates with UV-Vis data and evidences the formation of PANI-ES. The transparency, homogeneity and optical quality of the films are also clearly verified in the presented digital photographs.

**Figure 3:**
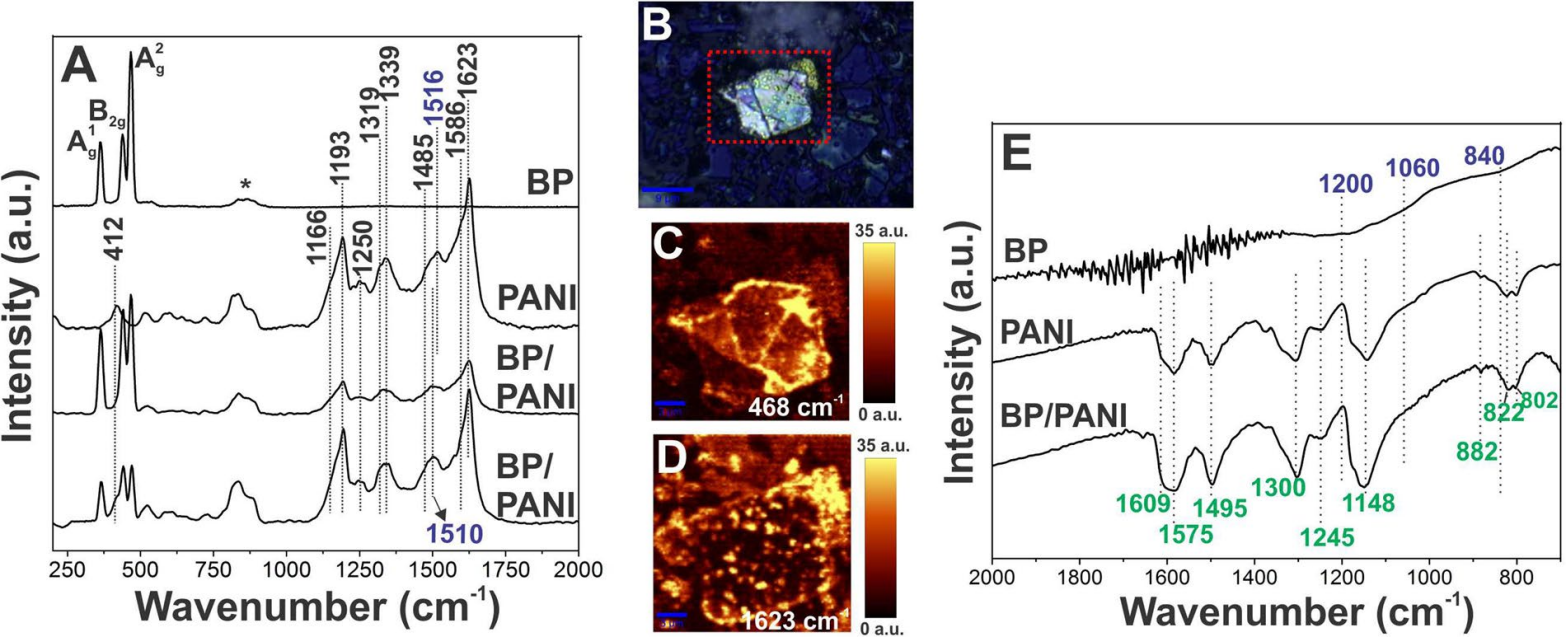
(**A**) Raman spectra of BP, PANI and BP/PANI (λ = 532 nm), (**B**) optical image of the flake used to obtain the (**C**,**D**) Raman mapping images of BP/PANI corresponding to the intensity of the bands of BP and PANI, respectively, and (**E**) FTIR spectra of BP, PANI and BP/PANI deposited over a ZnSe crystal window.

In the following, the samples were characterized by Raman spectroscopy allied to a confocal microscope. The spectra obtained for BP, PANI and BP/PANI are presented in Fig. 3, along with its optical image. Figure 3A exhibit two representative Raman spectra of the nanocomposite (collected at two different regions of the sample) along with the spectra of the control samples. It is worth highlighting the coexistence of the modes of the two components in the nanocomposite spectra, which confirms the successful synthesis of a nanocomposite between BP and PANI. The presence of BP is confirmed by the presence of the bands referring to the active modes ${{\rm{A}}}_{1{\rm{g}}}$, ${{\rm{B}}}_{2{\rm{g}}}$ and ${{\rm{A}}}_{2{\rm{g}}}$, already described in the previous section. Moreover, it is possible to detect the characteristic spectrum of the polymer in its conducting form, as described previously, represented by the bands presented in detail in Table S1 ^32–34^. Overall, there are no significant changes in the spectra of both BP and PANI in comparison with the nanocomposite, except for the 1516 cm^−1^ band of neat PANI that is shifted to 1510 cm^−1^.

Figure 3C,D show the Raman mappings images obtained from the indicated flake of Fig. 3B and refer to the intensities of the bands centered at 468 and 1623 cm^−1^, related to BP and PANI, respectively. The different intensities of the ${{\rm{A}}}_{2{\rm{g}}}$ band throughout the mapped flake in Fig. 3C is possibly related to the flake thickness, thereby are higher for thicker regions^35^. Furthermore, Fig. 3D shows that the polymer is well-distributed over the surface of BP flakes as suggested by SEM images. Figure S4 shows further Raman mappings of the sample which agree with the homogenous distribution and favorable interaction on the nanocomposite.

Seeking for more evidence of the BP/PANI film synthesis, the samples were deposited on ZnSe crystals windows and characterized by FTIR. The spectra obtained for BP, PANI and BP/PANI are shown in Fig. 3E. For BP spectrum, Fig. 3 exhibits the presence of the same bands presented in Fig. S1B, which refer to modes resulting from the phosphorus oxidation process^27–29^. In addition, the higher intensity of these modes, allied to the higher noise related to the sample moistening^30^, imply that the process for the film formation actually accelerates PO_x_ formation, as expected. The typical FTIR bands of PANI in the ES form are detectable in both neat polymer and nanocomposite, corroborating with Raman conclusions. The attributions of the bands are showed in Table S2. Overall, it is observed that the BP/PANI spectrum presents the unaltered bands of the pure polymer, with no significant variations of position or intensity. In addition, bands related to PO_x_ structures were not found, suggesting that in fact PANI acts as an efficient protector against degradation of BP. Thus, the next session describes a thorough stability study of the bare and the capped material, against degradation, when exposed to ambient conditions.

### Stability study of the BP-based nanocomposite

Considering that the main focus of the present study was to evaluate if PANI can efficiently protect BP against oxidation in a novel nanocomposite thin film, we pursued a detailed stability study. For this purpose, we monitored daily (for 60 days) the BP degradation when incorporated into the nanocomposite, by Raman and FTIR spectroscopies.

**Figure 4:**
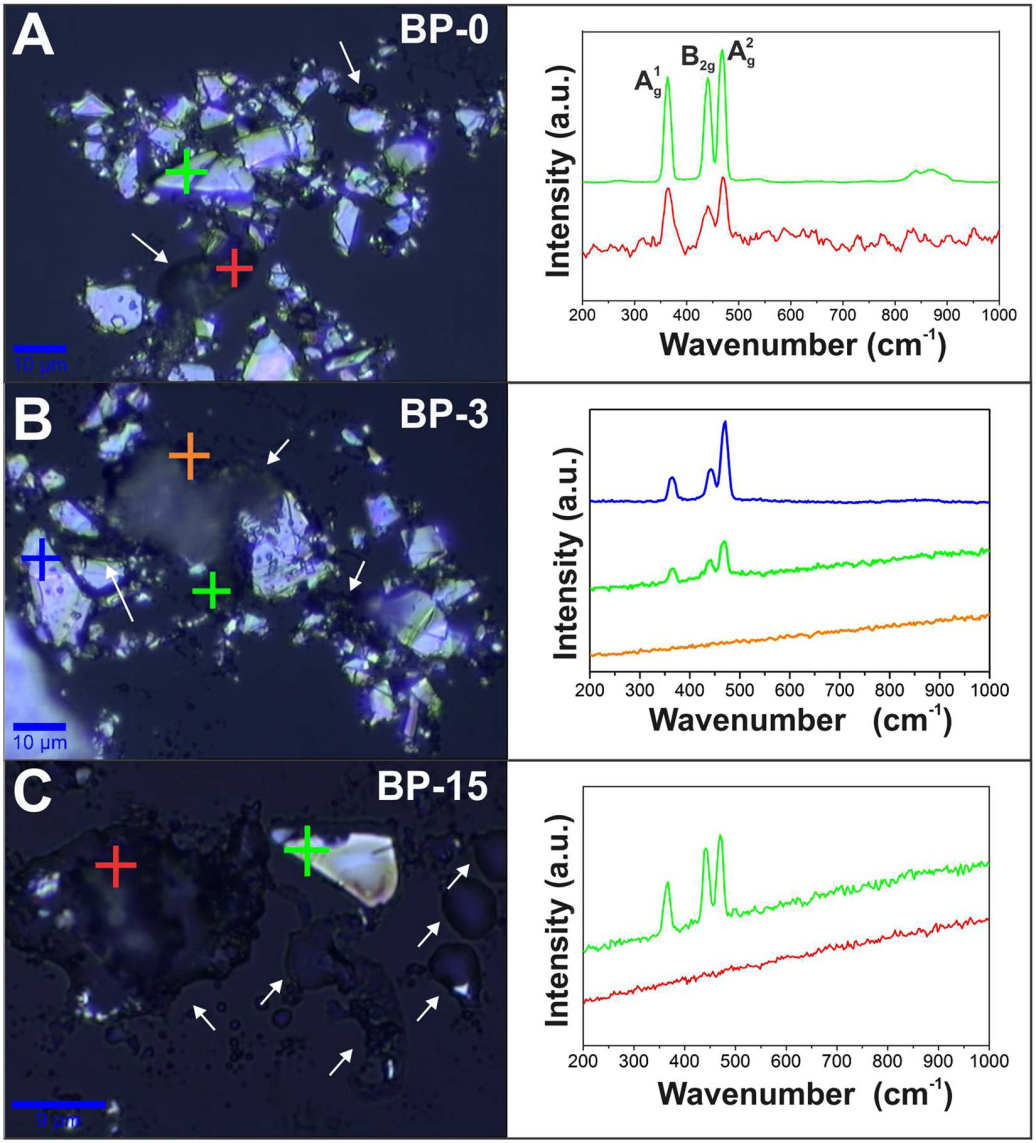
Optical imaging and representative Raman spectra (λ = 532 nm) of BP deposited over glass substrate with (**A**) 0 day, (**B**) 3 days and (**C**) 15 days of exposure to ambient conditions.

Figure 4 shows the stability results obtained from Raman analysis when monitoring the uncapped material (neat BP), where the spectra correspond to the regions indicated by the colored markings on the optical images. The sample BP-0 means that it was characterized right after vacuum drying, while BP-3 and BP-15 were taken after 3 and 15 days under ambient conditions, respectively. Analyzing the corresponding optical images, it is verified that the material presents distinct regions, in which some portions of the flakes are intact and other presents darker points (see arrows in Fig. 4A–C), related to the oxidation processes of BP. The spectra corresponding to optical image of Fig. 4A shows regions with the expected profiles of BP (green spectrum) and other with altered/noisy profiles (red), indicating the low intensity of the BP bands in the spectrum. In fact, the degradation of BP leads to the gradual decrease of the intensity of its Raman bands^5^ until the complete disappearance at the end of the decomposition process^7^. With the time following-up there is a clear increase in the darker portions of the sample (arrows in Fig. 4B,C), as well as a strong interference of a fluorescence effect on the baseline of the spectra, possibly due to the presence of impurities and/or BP degradation products^36^. Moreover, after only 3 days (Fig. 4B) it is no longer possible to detect BP in some regions (orange), which leads to almost complete degradation after 15 days (Fig. 4C) of exposition. Therefore, the results indicate that BP is significantly degraded under ambient conditions after solely 3 days.

**Figure 5:**
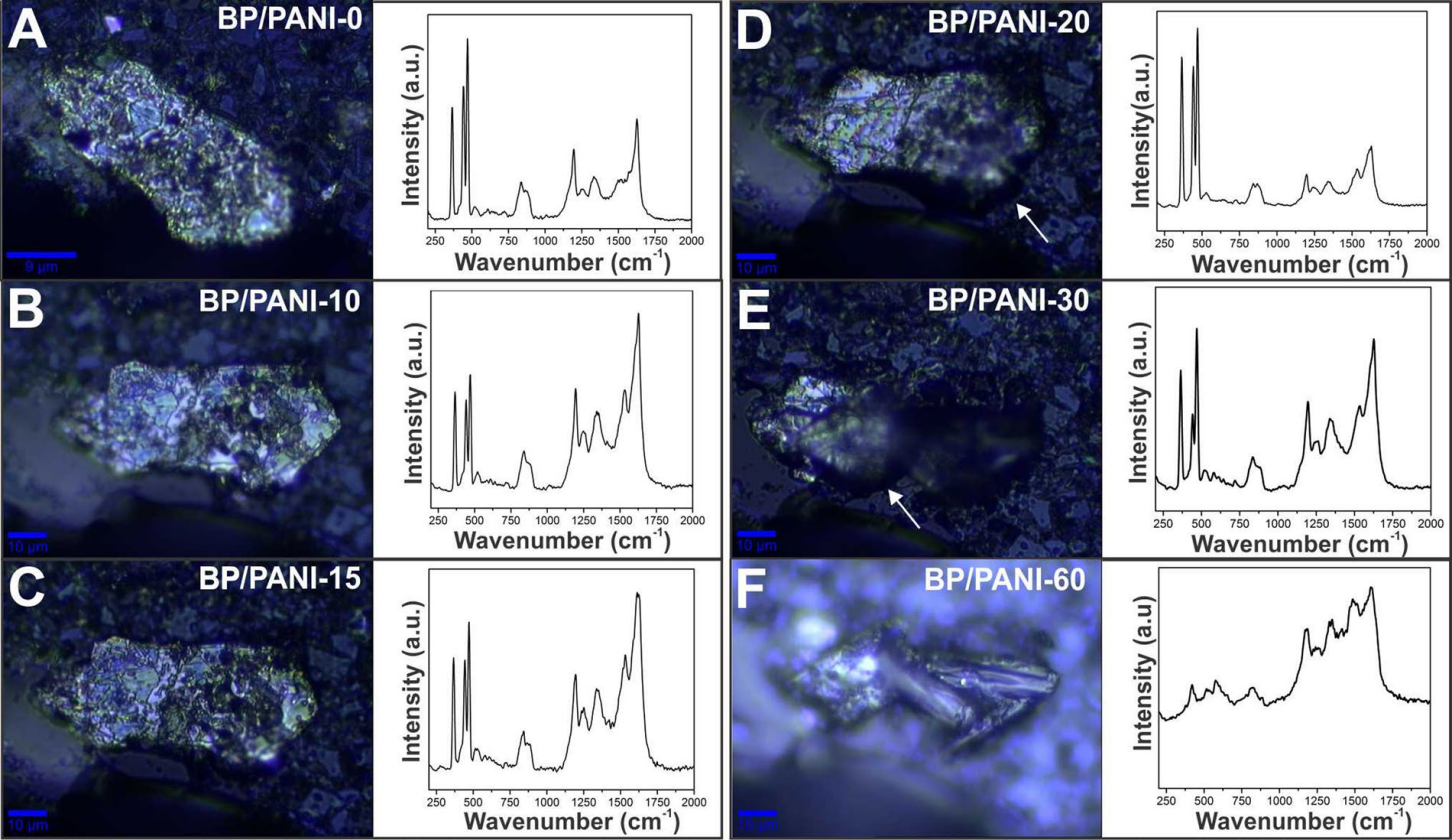
Optical images and Raman spectra (λ = 532 nm) of BP/PANI nanocomposite deposited over a glass substrate after (**A**) 0, (**B**) 10, (**C**) 15, (**D**) 20, (**E**) 30 and (**F**) 60 days of exposure to ambient conditions.

In the following, the stability of the BP/PANI nanocomposite was monitored analogously thus verifying the ability of the polymer to act as a barrier against BP oxidation. The optical images, along with their respective Raman representative spectra, are shown in Fig. 5 that were taken up to 60 days. Interestingly, it is possible to verify by the optical images that the material remains unchanged until about 20 days, when small changes start to be detected. Due to the absorption of moisture, the material undergoes physical changes, such as volume expansion^7^, reflecting on irregular surfaces that hinder the focus of the microscope (arrows in Fig. 5). Analyzing the corresponding spectra, we may note that even after 30 days of exposure, the Raman profiles remain unchanged and the typical BP bands are identified, despite the morphological changes in the flake. In fact, the complete vanish of BP bands is observed only with 60 days, as seen in Fig. 5F, that in contrast was with only 3 days of exposure for the neat BP (as showed in Fig. 4). Hence, besides obtaining a novel BP-based nanocomposite as thin film, results conclusively indicate that the polymeric component (PANI) can successfully act as a protective layer against BP degradation when exposed uninterruptedly to ambient conditions.

Figure S5 shows the higher frequency region of all spectra presented in Fig. 5. In general, by comparing the neat PANI spectrum with the nanocomposite for up to 30 days of exposure, no significant changes in the positions and intensities of the modes of vibration of the polymer are observed, indicating that the conductive nature of the material is conserved throughout this time. In contrast, the 60-day nanocomposite spectrum clearly shows changes in the positions and intensities of some vibration modes of the polymer, indicating the occurrence of possible chemical interactions between the polymer and the BP degradation products. The detailed discussion regarding the comparison between these spectra is depicted in SI.

**Figure 6:**
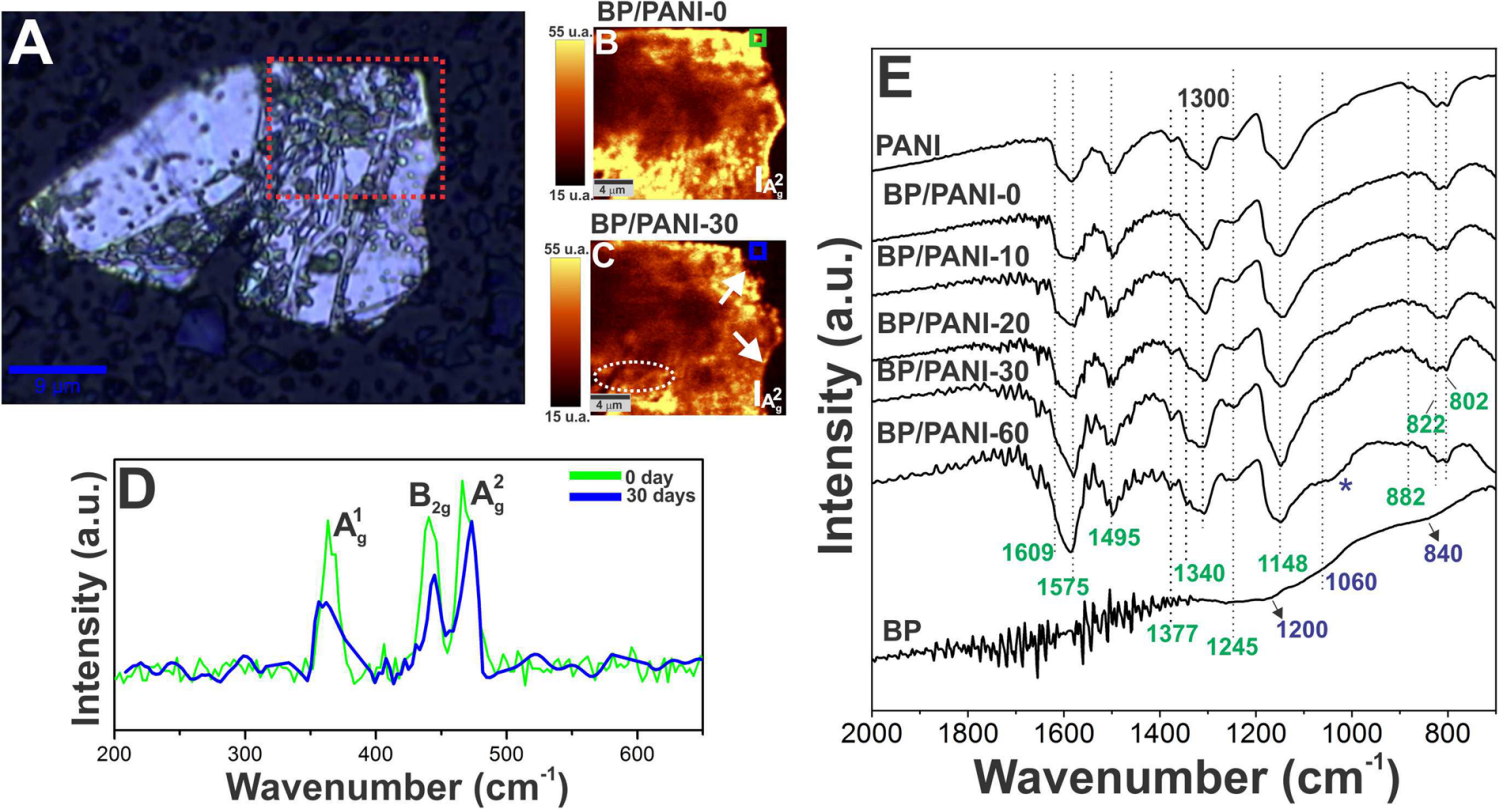
(**A**) Optical image of the flake used to obtain the (**B**,**C**) Raman mapping images of BP/PANI nanocomposite corresponding to the intensity of the ${{\rm{A}}}_{2{\rm{g}}}$ band of BP for the sample of 0 and 30 days in ambient conditions, (**D**) Raman spectra (λ = 532 nm) corresponding to the highlighted portions of BP/PANI-0 and BP/PANI-30 samples, and (**D**) FTIR spectra of BP, BP/PANI (from 0 to 60 days of exposure) and PANI.

Seeking a more comprehensive understanding of the nanocomposite stability, Raman mapping were also used and Fig. 6A–C shows the images obtained for the sample with 0 and 30 days of exposure. The images reported refer to the highlighted region indicated in Fig. 6A (see red square) and represent the intensity of the ${{\rm{A}}}_{2{\rm{g}}}$ band with 0 (BP/PANI-0) and 30 days (BP/PANI-30) of exposure. By comparing the obtained mappings it is notable that, although the band intensity decays in a few portions of the sample at 30 days (circle in Fig. 6C), the majority of the sample remains unchanged. Figure 6D shows the spectra collected at the edge regions of the material, indicated respectively by the green and blue squares on the maps. Observing the obtained profiles, an intensity quenching is detected and indicates that the degradation is seen to begin at the edges of the exfoliated material (arrows in Fig. 6B). In addition, the shift observed for the ${{\rm{B}}}_{2{\rm{g}}}$ and ${{\rm{A}}}_{2{\rm{g}}}$ bands may be associated with the decreasing in the number of sheets, that happens as a result of degradation process of the material^5^.

Finally, the nanocomposite stability was monitored by FTIR that are shown in Fig. 6E. The spectra indicate a series of positions and intensities changes of some modes of vibration in the polymer-related bands, which become more evident as the exposure time increases. The detailed discussion is presented in SI and the most important observation is a shoulder at around 1040 cm^−1^, indicated by the asterisk in Fig. 6E, that is observed for the sample with more time of exposure (>20 days) and gradually increases with time. Interestingly, a similar band at around 1060 cm^−1^ was observed for the bare BP, being an indication of oxidation (P-O stretch). Thus, as well as depicted by Raman data, FTIR spectra show that BP oxidation starts just from about 20 days of exposure when incorporated in the nanocomposite. Besides, in this case, the complete BP decomposition happens with about 60 days of exposure. Neat BP, however, starts to suffer oxidation processes with less than 3 days in ambient conditions, being completely degraded after 15 days, which indicates an increase in the material’s lifetime of about 600%.

As a final remark, it is important to notice that although it is well known that the stability of BP is strongly dependent on the flake thickness, both samples are comparable, once the exfoliated BP used for the beginning of aniline polymerization is exactly the same used for the stability studies of neat BP. As the polymer grows around and over the available BP flakes, they became protected by the polymer, and further agglomeration is hence avoided, which means that the thickness of the flakes remains in the nanocomposite exactly as initially present in the exfoliated neat BP. Also, it is well known that position of the BP Raman bands is dependent on the thickness of the flakes^5^. The Raman spectra of both neat BP and BP-PANI nanocomposite prepared here are exactly the same, with no bands shifts observable, which is an indicative that we are analyzing samples with comparable thickness, attesting that the monitoring was performed with comparable samples.

## Conclusions

Overall, we demonstrated that the L/L interfacial methodology is a versatile, cheap and simple way of preparing homogeneous and stable thin films of BP with PANI nanocomposite, not reported for phosphorus-based materials before. In addition, processing BP as thin film is difficult, evidencing the novelty of the nanocomposite obtained. Besides, previous studies of our research group^17–19^ have shown that the L/L interfacial route may lead to nanocomposite thin films with intimate contact between the components, which is interesting to obtain materials with synergistic features. Thus, BP/PANI nanocomposite may present promising enhanced properties when compared to the isolated materials, which would be suitable for technological perspectives.

In addition, it is important to highlight that here the materials were maintained under air-free atmosphere only during the exfoliation and polymerization processes. In the following, we have unprecedentedly employed spectroscopic techniques to evaluate the stability of the nanocomposite when exposed to ambient conditions for up to 60 days. Indeed, no glove boxes or extreme vacuum conditions were needed. To the best of our knowledge we report an innovative approach for obtaining stable BP that employs PANI as an efficient degradation inhibitor of BP. Moreover, the results herein clearly indicate an efficient coating of BP by PANI, what should justify the higher stability of BP in the nanocomposite.

By monitoring BP/PANI stability we could attest that the exfoliated material has its degradation kinetics drastically reduced when capped by the polymer. With almost 60 days of exposure it is still possible to detect the BP incorporated in the nanocomposite the uncapped BP, however, undergoes significant degradation in nearly 3 days.

Since BP applications are still limited by its high air-instability feature, this work not only provides insights on the synthesis of a novel nanocomposite, but also prints to an innovative route towards the development of BP-based devices, possible to be transferred as thin films into different kinds of substrates (plastic included), suitable to any desired field of application. Thus, we believe the present study is a breakthrough in the increasing area concerning BP properties, processing and applications, since it enlightens to new possibilities of designing, preparing and applying BP-based materials when compared to the existing methods. The properties and possible technological application of the BP/PANI nanocomposite thin film related here are currently under evaluation in our laboratory.

## Electronic supplementary material

Supplementary Information
