# Supplementary material for: Air stable black phosphorous in polyaniline-based nanocomposite
Source: Sci Rep. 2017 Aug 31;7:10165. doi: 10.1038/s41598-017-10533-5 (PMC5579270; doi:10.1038/s41598-017-10533-5)
Supplement: Supplementary file 1 — Supplementary Information [file 41598_2017_10533_MOESM1_ESM.doc]

**Air stable black phosphorous in polyaniline-based nanocomposite**

Jéssica E. S. Fonsaca*^a^*, Sergio H. Domingues*^b^*, Elisa S. Orth*^a^*, Aldo J. G. Zarbin*^a*^*

^a^ Department of Chemistry, Federal University of Parana (UFPR), CP 19032, CEP 81531-980, Curitiba, PR, Brazil.

^b^ Graphene and Nano-materials Research Center – Mackgraphe – Graphene and Nanomaterials Research Center, Mackenzie Presbyterian University, 01302-907 São Paulo, Brazil.

. **supplementary information**

| 1. Characterization of BP dispersion | S3 |
| --- | --- |
| 1.1. Raman and FTIR data | S3 |
| **Figure S1.** Exfoliated BP dispersed in acetonitrile and dried over glass (A) for Raman (ʎ = 532.02 nm) and over a ZnSe window (B) for FTIR measurements. The inset scheme indicates the different active vibrational modes in the BP crystal lattice. | S4 |
| 1.2. SEM images | S4 |
| **Figure S2.** SEM images of the obtained BP and elemental mapping by EDS performed in one flake, the inset presents the corresponding spectrum. | S5 |
| 2. UV-Vis spectra of BP-PANI nanocomposite | S5 |
| **Figure S3.** UV-Vis spectra of PANI and BP-PANI films deposited over quartz. | S5 |
| 3. Attributions of Raman bands of PANI (ʎ = 532.02 nm) | S6 |
| **Table S1.** Attribution of PANI bands of Raman spectrum of Figure 3A. | S6 |
| 4. Raman mapping of BP-PANI nanocomposite | S7 |
| **Figure S4.** (A) Optical image of BP-PANI film and (B-C) Raman mapping images corresponding to the intensity of the bands of BP and PANI, respectively. | S7 |
| 5. Attributions of FTIR bands of PANI. | S8 |
| **Table S2.** Attribution of PANI bands of FTIR spectrum of Figure 3E. | S8 |
| 6. Study of nanocomposite stability | S9 |
| 6.1. Raman analyses | S9 |
| **Figure S5.** Raman higher frequency region of all previous spectra presented in Figure 5 and, for comparison, PANI spectrum (ʎ=532.02 nm). | S10 |
| 6.2 FTIR analyses | S10 |
| Supplementary references | S10 |

1. **Characterization of BP dispersion**

**1.1 Raman and FTIR data**

**
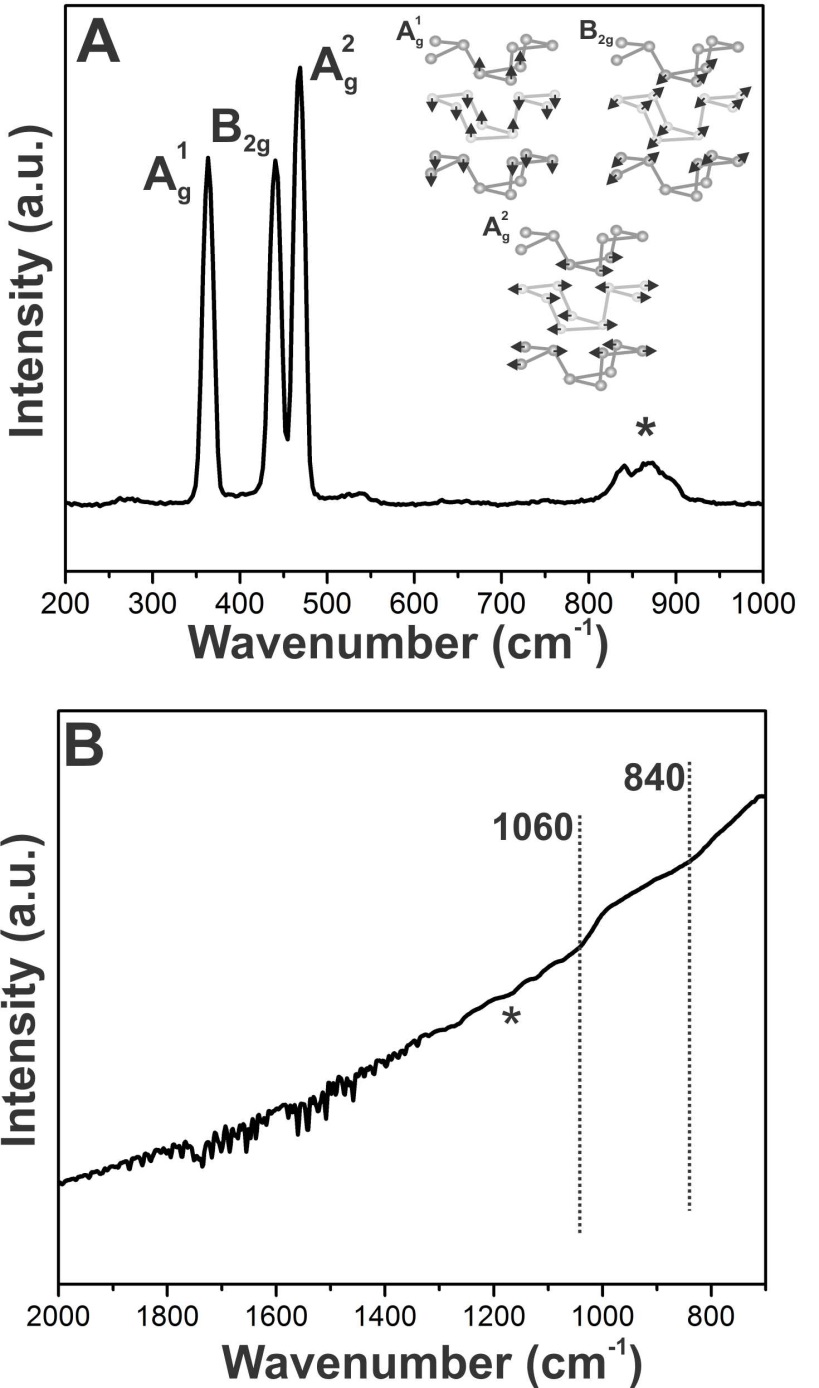
**

**Figure S1.** Exfoliated BP dispersed in acetonitrile and dried over glass (A) for Raman (ʎ = 532 nm) and over a ZnSe window (B) for FTIR measurements. The inset scheme indicates the different active vibrational modes in the BP crystal lattice.

**1.2 SEM images**

**
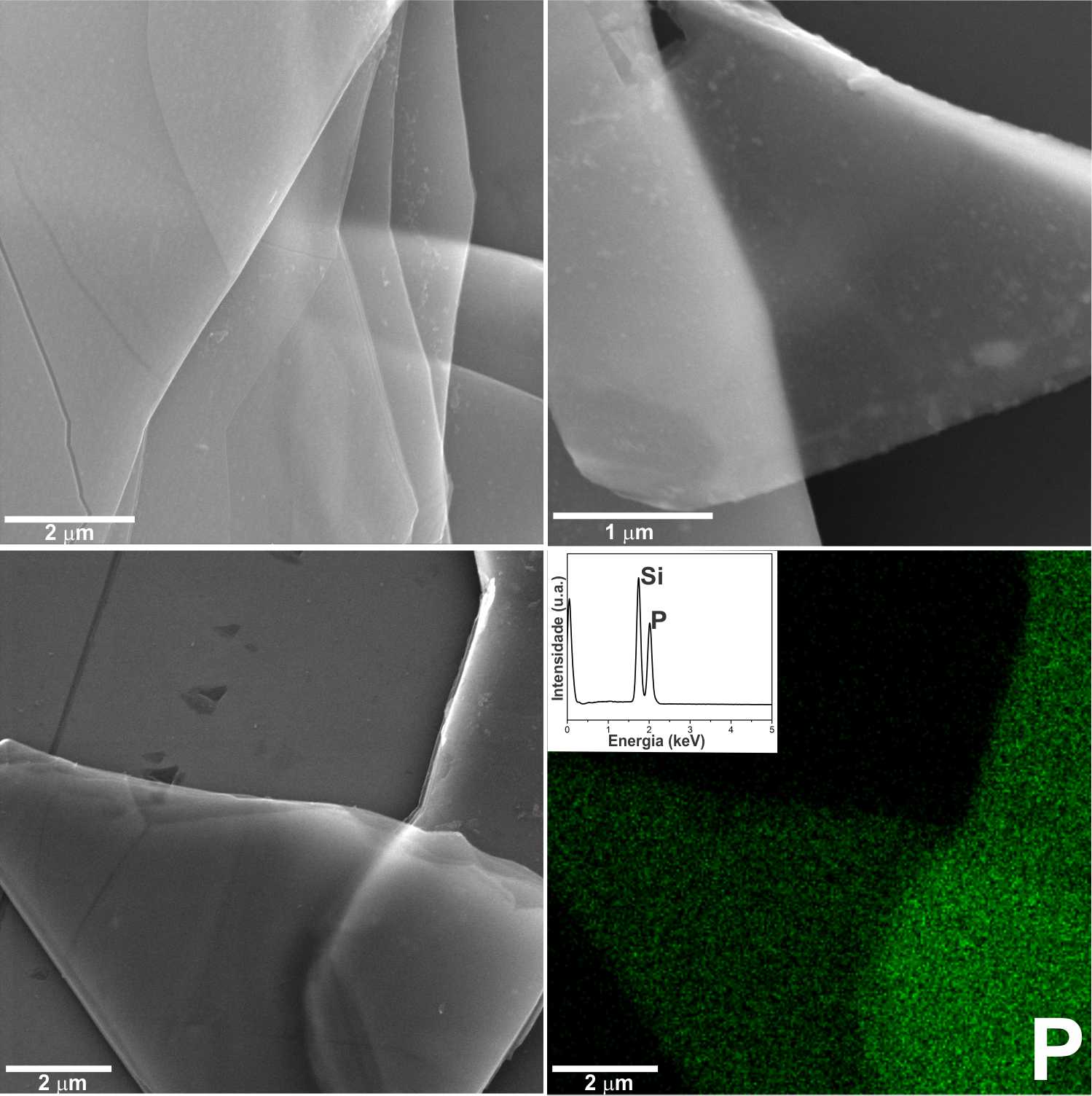
**

**Figure S2.** SEM images of the obtained BP and elemental mapping by EDS performed in a flake of the material, the inset presents the corresponding spectrum.

1. **UV-Vis spectra of BP-PANI nanocomposite**

**
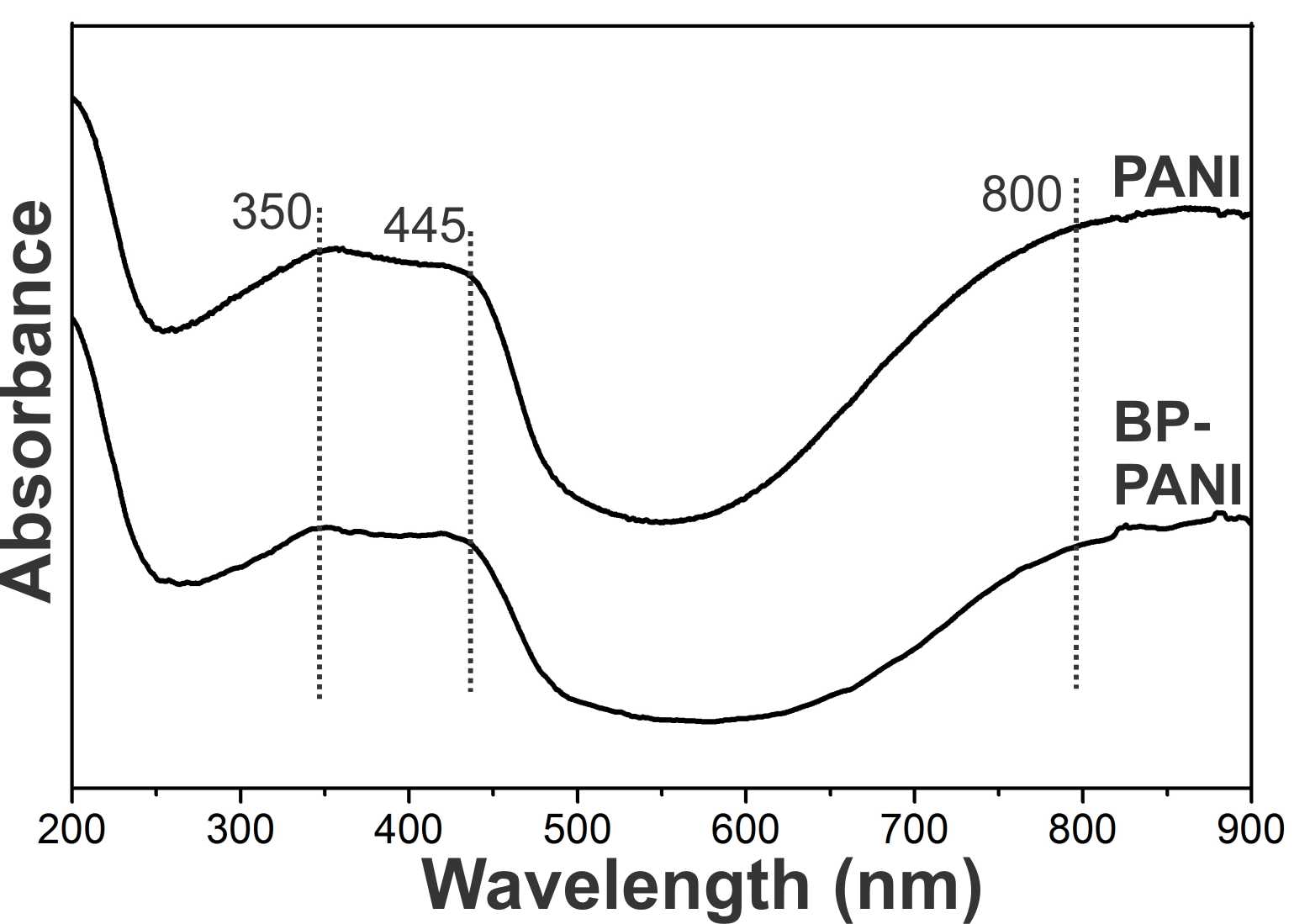
**

**Figure S3.** UV-Vis spectra of PANI and BP-PANI films deposited over quartz.

1. **Attributions of Raman bands of PANI (ʎ = 532 nm)**

**Table S1.** Attribution of PANI bands of Raman spectrum of Figure 3A.[^1-3^](#_ENREF_1)

| **Frequency (cm^-1^)** | **Attribution** |
| --- | --- |
| **412** | C-C bending |
| **1193** | Bending of benzenoid rings |
| **1250** | ν(C-N) benzene diamine units |
| **1319 and 1339** | ν(C-N^+^) of the polaron radical cation |
| **1485** | ν(C=N) of the quinoid nonprotonated di-imine units |
| **1510-1516** | ν(C=N) of the quinoid protonated di-imine units |
| **1586** | ν(C=C) of the quinoid rings |
| **1623** | ν(C-C) of the benzene rings |

1. **Raman mapping of BP-PANI nanocomposite**

**
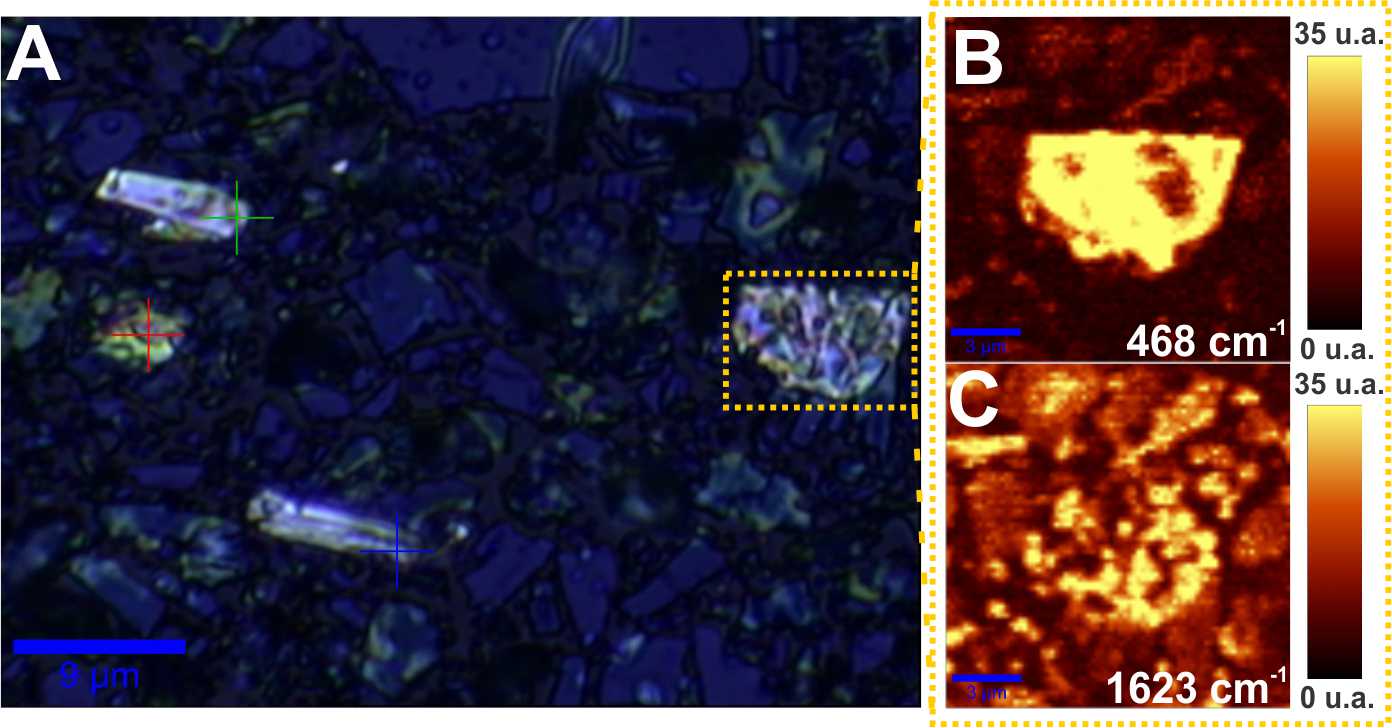
**

**Figure S4.** (A) Optical image of BP-PANI film and (B-C) Raman mapping images corresponding to the intensity of the bands of BP and PANI, respectively.

1. **Attributions of FTIR bands of PANI**

**Table S2.** Attribution of PANI bands of FTIR spectrum of Figure 3E.[^4-5^](#_ENREF_4)

| **Frequency (cm^-1^)** | **Attribution** |
| --- | --- |
| **1609** | νC=C in polarons |
| **1575** | Strecthing of the quinoid ring |
| **1495** | Strecthing of the benzenoid ring |
| **1300** | νC-N in delocalized polarons |
| **1245** | νC-N^+*^ in the polaron structure |
| **1148** | Vibration mode of –NH^+^= structure |
| **882 and 822** | δC-H out of the plane |
| **802** | δC-H out-of-plane on substituted 1,4 rings |

1. **Study of nanocomposite stability**

**6.1. Raman analyses**

**
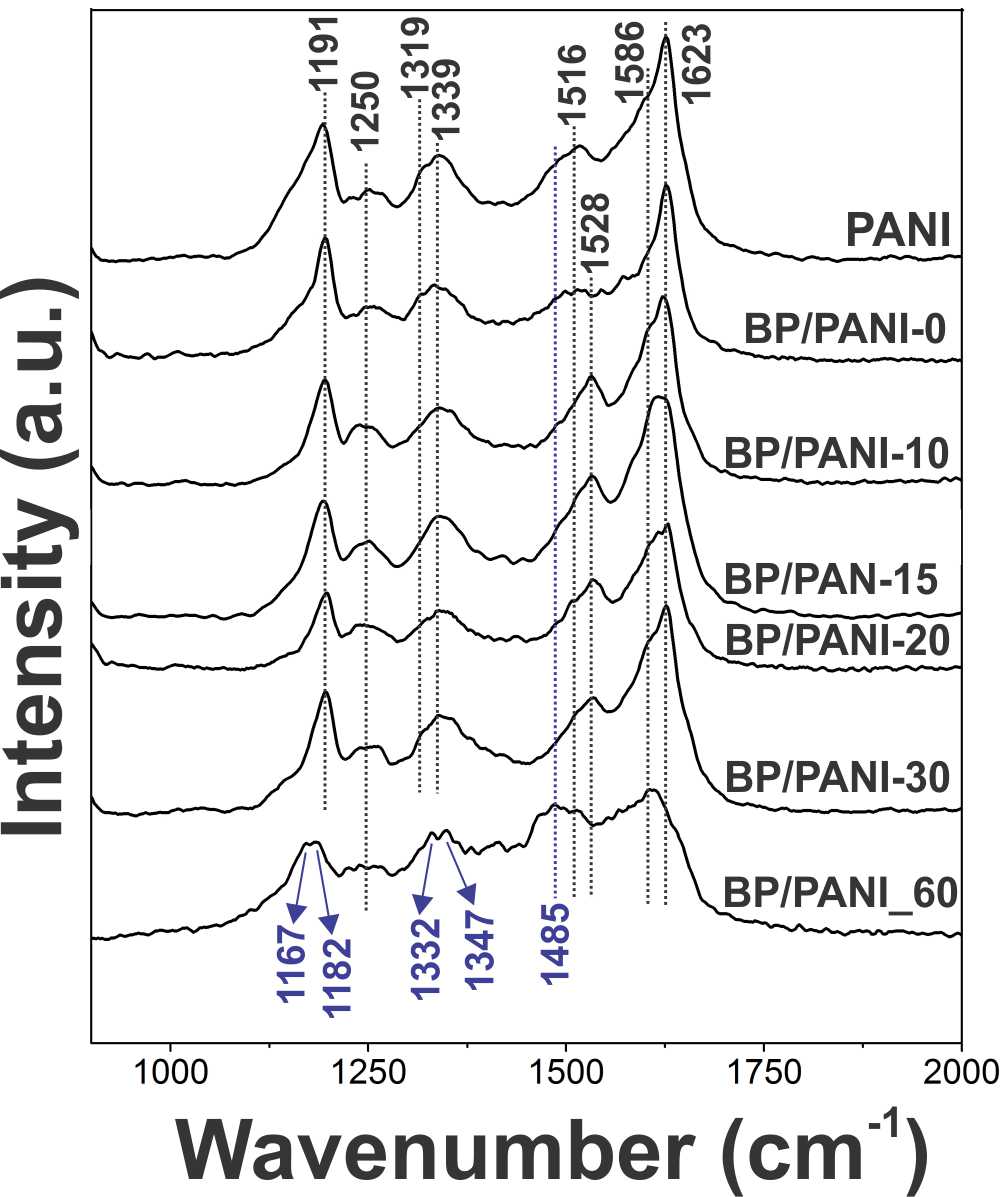
**

**Figure S5.** Raman higher frequency region of all previous spectra presented in Figure 5 and, for comparison, PANI spectrum (ʎ=532 nm).

The polymer bands located at 1165-1191 cm^-1^ in **Figure S5** indicate the overall oxidation state of the polymer or the kind of the carrier (polarons and bipolarons) present.[^1^](#_ENREF_1) Which means that in the conductive form, this vibration is found at approximately 1189 cm^-1^ for the polaronic reticulum (benzenoid rings) and 1165 cm^-1^ for the bipolar form (quinoid rings), with shift to the more reduced or oxidized materials.[^1^](#_ENREF_1) From **Figure S5**, it is possible to verify that the BP degradation with about 60 days leads to the increase of the intensity of the band at 1167 cm^-1^ (relative to the band at 1165 cm^-1^), in other words, leads to the increase of bipolaronic type carriers, which means that the polymer is less conductive. Although the mode at 1250 cm^-1^ is also related to doping, its low intensity makes it difficult to observe trends.

The wide bands at 1319-1340 cm^-1^ are known as polaronic bands and reflect the number of carriers. The coalescence of these bands, as observed in the 0-30-day exposure spectra, indicates more extended polymer chains and the presence of a single carrier type.[^6-7^](#_ENREF_6) Thus, its splitting, as observed at 60 days, is associated with different types of carriers, with the values at 1348 cm^-1^ referring to delocalized polarons, while the values at 1329 cm^-1^ correspond to the presence of two polarons per repeating unit of the polymer (polar reticulum).[^8^](#_ENREF_8) According to the literature, the separation of these bands suggests the formation of a less conductive PANI.[^2^](#_ENREF_2) The shoulder, centered at 1380 cm^-1^, only observed in the 60-day nanocomposite, is related to the formation of chains containing tertiary nitrogen, in structures similar to oxazine and phenazine rings. The absence of this band in the nanocomposite spectra with less time of exposure evidences less reticulated chains.

Also, it may be noted that the intensity of the band at 1485 cm^-1^ increases with respect to the band centered at 1516-1528 cm^-1^, indicating that a selective interaction might be taking place between quinoid ring of the doped polymer and the degradation product,[^10^](#_ENREF_10) directly affecting the transport properties of the material. Furthermore, the higher definition of the band centered at 1623 cm^-1^ corroborate with the less polaronic structure of the polymer.

**6.2. FTIR Analyses**

From **Figure 6E** depicted in the manuscript it is possible to verify that the most significant changes are observed from 20 days of exposure, as observed with Raman spectroscopy. Initially, it may be observed the 1575 cm^-1^ band becoming more defined with respect to the shoulder at 1609 cm^-1^, which indicates the stabilization of the quinoid rings in the polymer structure[^5^](#_ENREF_5) over time, possibly by the BP degradation products, as mentioned above.

Similar changes can also be observed in other polymer-related bands, such as the periodic increase of some vibrational modes: (i) at 1540 cm^-1^, referring to the C-C stretch in quinoid rings and (ii) at 1340 and 1377 cm^-1^, attributed to C-N stretches in the vicinity of the quinoid rings.[^5^](#_ENREF_5) Possibly, this increase in the quinoid (or bipolaronic) modes occurs due to the interaction of the PO_x_ species with the more delocalized carriers of the polymer,[^10^](#_ENREF_10) also indicating the stabilization of the quinoid rings in the PANI structure.

**Supplementary references**

1. Bernard, M. C.; Hugot-Le Goff, A., Quantitative characterization of polyaniline films using Raman spectroscopy: I: Polaron lattice and bipolaron. *Electrochim. Acta* **2006,** *52* (2), 595-603.

2. Mažeikienė, R.; Tomkutė, V.; Kuodis, Z.; Niaura, G.; Malinauskas, A., Raman spectroelectrochemical study of polyaniline and sulfonated polyaniline in solutions of different pH. *Vib. Spectrosc* **2007,** *44* (2), 201-208.

3. Harada, I.; Furukawa, Y.; Ueda, F., Vibrational spectra and structure of polyaniline and related compounds. *Synth. Met.* **1989,** *29* (1), 303-312.

4. Trchová, M.; Šeděnková, I.; Konyushenko, E. N.; Stejskal, J.; Holler, P.; Ćirić-Marjanović, G., Evolution of Polyaniline Nanotubes:  The Oxidation of Aniline in Water. *The Journal of Physical Chemistry B* **2006,** *110* (19), 9461-9468.

5. Trchová, M.; Stejskal, J., Polyaniline: The infrared spectroscopy of conducting polymer nanotubes (IUPAC Technical Report). *Pure Appl. Chem.* **2011,** *83* (10), 1803-1817.

6. Silva, J. E. P. d.; Faria, D. L. A. d.; Torresi, S. I. C. d.; Temperini, M. L. A., Influence of Thermal Treatment on Doped Polyaniline Studied by Resonance Raman Spectroscopy. *Macromolecules* **2000,** *33* (8), 3077-3083.

7. Cochet, M.; Louarn, G.; Quillard, S.; Buisson, J. P.; Lefrant, S., Theoretical and experimental vibrational study of emeraldine in salt form. Part II. *J. Raman Spectrosc.* **2000,** *31* (12), 1041-1049.

8. Bernard, M. C.; Goff, A. H.-L.; Joiret, S.; Arkoub, H.; Saidani, B., Influence of the nature of substituent on the charge mechanisms in substituted polyanilines (SPANI, POMA) studied by Raman and optical spectroscopies. *Electrochimica Acta* **2005,** *50*, 1615-1623.

9. Šeděnková, I.; Trchová, M.; Stejskal, J., Thermal degradation of polyaniline films prepared in solutions of strong and weak acids and in water - FTIR and Raman spectroscopic studies. *Polymer Degradation and Stability* **2008,** *93*, 2147-2157.

10. Cochet, M.; Maser, W. K.; Benito, A. M.; Callejas, M. A.; Martinez, M. T.; Benoit, J.-M.; Schreiber, J.; Chauvet, O., Synthesis of a new polyaniline/nanotube composite: "in-situ" polymerisation and charge transfer through site-selective interaction. *Chem. Commun.* **2001,** (16), 1450-1451.
